# A New Method of Detecting Pulmonary Nodules with PET/CT Based on an Improved Watershed Algorithm

**DOI:** 10.1371/journal.pone.0123694

**Published:** 2015-04-08

**Authors:** Juanjuan Zhao, Guohua Ji, Yan Qiang, Xiaohong Han, Bo Pei, Zhenghao Shi

**Affiliations:** 1 College of Computer Science and Technology, Taiyuan University of Technology, Taiyuan 030024, China; 2 College of Computer Science and Engineering, Xi’an University of Technology, Xi’an 710048, China; University of Nebraska Medical Center, UNITED STATES

## Abstract

**Background:**

Integrated ^18^F-fluorodeoxyglucose positron emission tomography/computed tomography (^18^F-FDG PET/CT) is widely performed for staging solitary pulmonary nodules (SPNs). However, the diagnostic efficacy of SPNs based on PET/CT is not optimal. Here, we propose a method of detection based on PET/CT that can differentiate malignant and benign SPNs with few false-positives.

**Method:**

Our proposed method combines the features of positron-emission tomography (PET) and computed tomography (CT). A dynamic threshold segmentation method was used to identify lung parenchyma in CT images and suspicious areas in PET images. Then, an improved watershed method was used to mark suspicious areas on the CT image. Next, the support vector machine (SVM) method was used to classify SPNs based on textural features of CT images and metabolic features of PET images to validate the proposed method.

**Results:**

Our proposed method was more efficient than traditional methods and methods based on the CT or PET features alone (sensitivity 95.6%; average of 2.9 false positives per scan).

## Introduction

Lung cancer is one of the most common causes of cancer-related deaths worldwide [1]. In early stages, lung cancer appears predominantly as solitary pulmonary nodules (SPNs). Early detection and treatment for SPNs is necessary for survival and PET and CT are commonly used to detect and characterize SPNs. However, the large image numbers produced by PET and CT scanning increases workload and increases chances of misdiagnoses and missed diagnosis. Computer-aided detection (CAD) systems could help physicians improve detection efficiency and accuracy of identifying lung nodules and these systems have been instrumental for early detection of lung cancer [2, 3]. The evolution of medical image processing and artificial intelligence techniques has provided excellent opportunities to improve CAD efficacy for detecting SPNs.

Lung segmentation is often a preprocessing step for nodule detection with CT imaging. Guo’s group used an iterative neutrosophic methodology to segment the lung in thoracic CT images [4]. Wei and coworkers used several methods, including optimal iterative threshold, three-dimensional connectivity labeling, and three-dimensional region growing for initial segmentation of lung parenchyma (based on improved chain code) and used Bresenham’s algorithm to repair the lung parenchyma [5]. Darmanayagam and colleagues segmented the lung in three steps [6] as follows: segmentation was first performed using an iterative threshold, and this was followed by morphological operations to extract shape features of both lungs. Then, a multilayer feed-forward neural network was used to determine whether the segmented lung parenchyma was complete. Three-dimensional images of both lungs were then generated. In general, lung segmentation methods can be divided into threshold-, region-, and mathematical-morphology-based methods. Threshold-based methods offer fast calculations and implementation ease. With region-based image segmentation, a specific image region can be acquired, and mathematical methods can guarantee accurate segmentation results.

Various CAD schemes for SPNs based on CT images have been proposed recently. Okada’s group researched the extraction of ground glass nodules in high-resolution CT [7], proposing a semiautomatic method based on CT image attenuation values to differentiate ground-glass opacity and solid-type lung cancers. Yan’s clinic investigated incremental effects of using high-performance CAD systems to detect solitary pulmonary nodules in chest radiographs [8]. They found that observer performance could be greatly improved when CAD systems were used as second readers, especially for small nodules and nodules occluded by ribs. Suárez-Cuenca and coworkers developed a CAD system to detect pulmonary nodules on thin-slice helical computed tomography (CT) images [9], including using an iris filter to discriminate between nodules and false-positive findings. Suspicious regions were characterized with features based on iris filter output, gray level, and morphological features extracted from CT images. Functions calculated using linear discriminant analysis (LDA) were used to reduce false positives. The system was trained and evaluated using two completely independent data sets. Results for a test set, evaluated with free-response receiver operating characteristic (ROC) analysis, yielded a sensitivity of 80% at 7.7 false-positives per scan. Opfer’s group proposed a method involving validation with Lung Image Database Consortium (LIDC) data sets [10]. CAD system performance was analyzed using the virtual mode of multiple free response ROC curves (FROC) for different lower thresholds of the nodule diameter. Data show a detection rate of 89%, and the median false positive rate of the findings per patient was 87.3%. Messay and colleagues proposed a novel CAD system for detecting pulmonary nodules in thoracic CT imagery [11] and this method was validated with a publicly available database. For this method, training and tuning of all modules was performed using a separate and independent dataset provided by the University of Texas Medical Branch (UTMB). Data confirm that the proposed front-end detector/segmentor could detect 92.8% of all nodules in the LIDC/testing dataset.

Genetic algorithms (GA) have been applied to pulmonary nodule detection. Dehmeshki’s group proposed a shaped-based GA template-matching (GATM) method to detect nodules with spherical elements [12]. The method was validated with a clinical dataset of 70 thoracic CT scans containing 178 nodules, and 160 nodules were correctly detected. Choi and colleagues proposed a detection method based on a genetic-programming-based (GP) classifier suitable for detecting nodules because of its flexibility and power [13]. Data show that our proposed method significantly reduced false-positives in nodule candidates and offered a 94.1% sensitivity with 5.45 false-positives per scan.

The accuracy and efficiency of lung nodule detection based on CT images does not meet clinical needs. Thus, detection methods based on PET/CT are proposed. Takahashi and colleagues focused on the commonly-used combined modality of PET/CT [14]. Because CT scanning generates attenuation-coefficient-based images of anatomy and PET depicts tissue metabolism based on uptake of radiolabeled tracers such as fluorodeoxyglucose, both of these methods together may improve detection accuracy. Zhang’s group reported that PET/CT indicates precise localization of focal abnormalities [15] and they used a segmentation method based on dynamic threshold to generate a CAD method for pulmonary nodule detection in PET/CT examinations.

However, because diagnostic efficacy of SPNs with PET/CT is challenging, we propose a novel lung nodule detection method using PET/CT and an improved watershed algorithm. Compared with the existing methods, our proposed method detects SPNs with more sensitivity and differentiates malignant and benign SPNs with fewer false-positives.

## Materials and Methods

### 2.1 Materials

#### 2.1.1. Ethics statement

This study was approved by the institutional review board (IRB) of the Coal Center Hospital in Shanxi. The study was conducted in accordance with the hospital’s ethics requirements. Informed consent was obtained from all patients for being included in the study.

#### 2.1.2. Study population

Among subjects who underwent SPN detection with PET/CT, a review was undertaken for patients who underwent surgery from January 2010 to January 2013 at the Coal Center Hospital in Shanxi. All data can be accessed at http://pan.baidu.com/s/1bnCNQij; the extraction password is bf2e. All patients who underwent neoadjuvant chemotherapy and patients with bulky mediastinal node metastases before thoracotomy were excluded. The remaining 219 consecutive patients with histologically proven SPN were staged with integrated PET/CT diagnoses. Of these 219 participants, 120 patients were confirmed to have 120 SPNs using plain/enhanced CT scans and the remaining 99 patients had inflammation.

#### 2.1.3. Integrated PET/CT scan

PET/CT was performed using a Discovery* PET/CT 610 system (GE Healthcare) with a preset slice thickness of 0.35 mm. All patients fasted for at least 6 h prior to PET/CT scanning and only glucose-free water was allowed. An intravenous injection of 3.7 MBq of ^18^F-FDG/kg was administered and patients rested for 60 min before scanning. PET/CT data were obtained from patients in the supine position. Emission images were acquired after CT scanning, and an emission scan was performed in 8–10 bed positions with 1 min per step. CT image resolution was 256 x 256 and that of the PET images was 128 x 128. Tumor ^18^F-FDG uptake was visually compared to surrounding tissue in areas devoid of prominent artifacts. A chest radiologist with 18 years of experience in CT interpretation and a nuclear medicine physician with 4 years of PET/CT interpretation jointly evaluated the integrated PET/CT images. The abnormal focal ^18^F-FDG uptake that accompanied corresponding anatomic alterations was considered indicative of metastasis. All SPNs with abnormal ^18^F-FDG uptake in the extrathoracic regions were considered metastatic unless they had high attenuation over 70 HU or benign calcification with unenhanced CT. Nodal uptake with a SUV_max_ >2.5 was interpreted as positive. All integrated PET/CT imaging was performed within 4 weeks of surgery

### 2.2 Proposed method of detection

**Fig 1:**
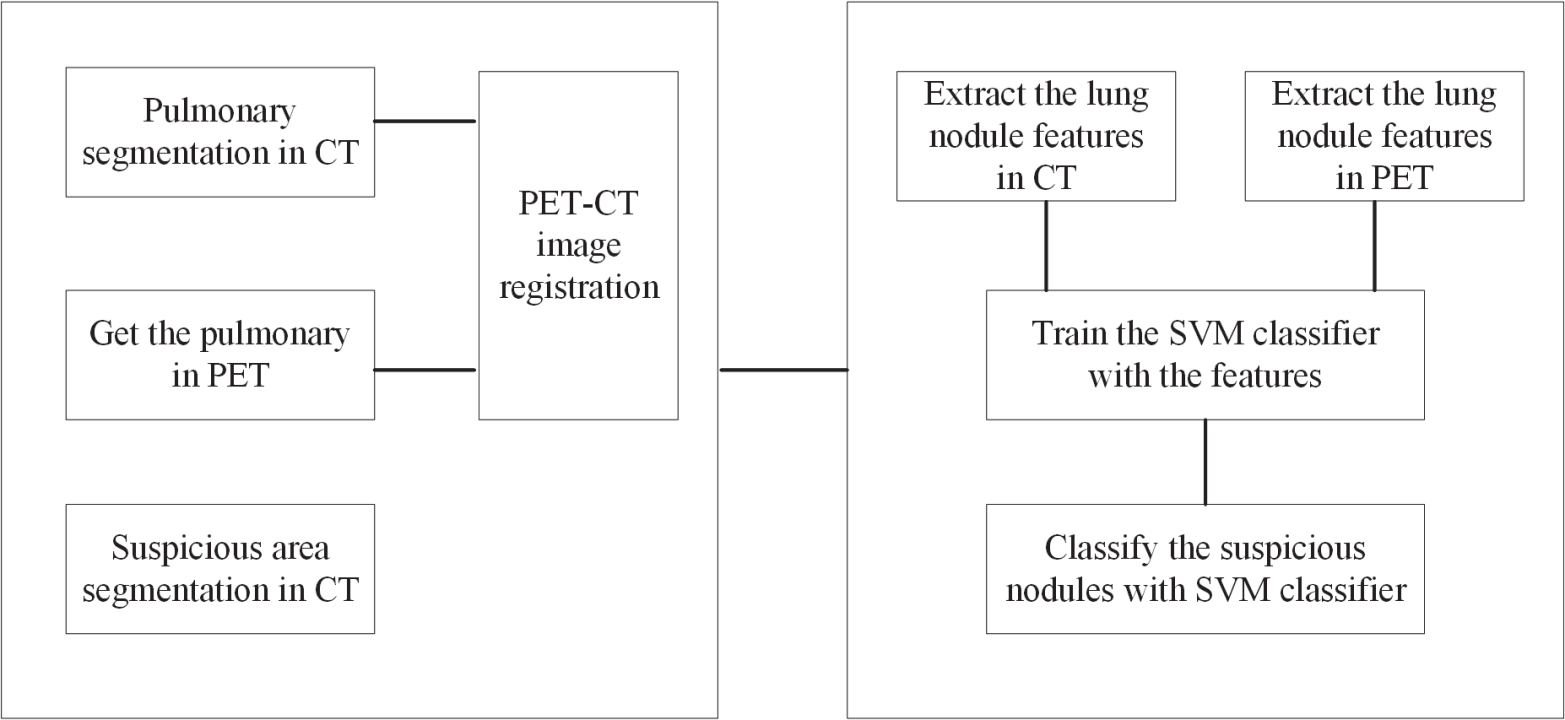
Diagram of the CAD system.

Fig 1 shows a block diagram of the CAD system based on the proposed method. The modules of the CAD system were processed consecutively.

Step 1: Lung segmentation in CT;

Step 2: PET/CT image registration based on mutual information;

Step 3: Segmentation of SPNs based on improved watershed algorithm;

Step 4: Feature extraction of solitary pulmonary nodules on PET/CT;

Step 5: Classification of SPNs based on support vector machine (SVM).

#### 2.2.1. Lung segmentation in CT

The lung is a very complex organ and lung segmentation involves many tissues, such as the bronchi, pulmonary arteries, and capillaries. Many segmentation methods have been used in medical image processing but considering that our concern was chiefly a solitary pulmonary nodule in the parenchyma, the lung was segmented using a dynamic threshold segmentation method based on CT images.

CT scan numbers are related to the attenuation coefficient of diagnostic X-rays and actually reflect electron densities of individual tissues. Scan numbers also define the position of an ROI. To reduce SPN detection complexity with PET, suspicious regions are usually first identified in CT images. Dividing boundaries of connected tissues is difficult with mono-threshold segmentation, even though there are significant differences in the gray level between the parenchyma and other tissues of the body [16, 17]. For this work, non-body parts were separated from lung images. The principle of the segmentation of the parenchyma based on the dynamic threshold segmentation method is as follows:

Step 1: Determine the initial value of threshold *p*_*i*_ (*i = 0*) according to the experience;

Step 2: Render lung image binary based on the preset threshold *p*_*i*_, the pixel values of the parenchyma are 0;

Step 3: Calculate average gray level of the parenchyma *p*_*i*_ and other tissues *p*_*t*_;

Step 4: Set *i* = *i*+1, re-calculate the threshold *p*_*i*_ according to Eq (1)

$$p_{i}=\frac{p_{i}+p_{t}}{2}$$ (1)

Step 5: If |*p*_*i*_ − *p*_*i*−1_|>*σ*(*σ*>0), jump to Step 2, otherwise proceed to step 6;

Step 6: If the gray value of the image is larger than *p*_*i*_, set *p*_*i*_ = 255 as the image background; otherwise, set *p*_*i*_ = 0 as the parenchyma.

#### 2.2.2. PET-CT image registration based on mutual information

To accurately identify SPNs, after pulmonary parenchyma area segmentation, CT and PET images were registered. SUV_max_≥2.5 of the suspicious area in the PET image is considered a criterion for distinguishing malignant or benign SPN. After CT and PET images registration, the spatial position of both image types can be confirmed and suspicious areas in CT images can be eliminated as much as possible. This process can greatly reduce the time required for subsequent segmentation and offer a foundation for feature extraction.

Mutual information measurement has been successfully applied to voxel-based multimodal medical image registration, which is an automatic measure suitable for multimodal registration [18]. The mutual information, the correlation-ship degree of the corresponding feature points, is largest when the two images reach consensus in spatial position after registration. This is an important concept in information theory as it measures the statistical dependence between two variables, i.e. the information that one variable carries about the other. This was first proposed as a method of registering medical images in 1995, by Viola and Wells and separately by Collignon [19, 20]. At this moment, mutual information is accepted by many researchers as one of the most accurate and robust retrospective registration methods.

Image registration aims to geometrically match two or more images of the same scene. Images taken at different times from different viewpoints and by different sensors for structure/target localization, difference detection, and other purposes are being increasingly used in the medical imaging [21].

In this study, PET and CT images of the dataset selected here were from the same slice of the same subject, so only rigid transformation needed to be considered.

Given two images *A* and *B*, the definition of the mutual information *I* (*A*, *B*) of these images is as follows:

I(A,B)=H(A)+H(B)−H(A,B) (2)

Here, *H* (*A*) and *H* (*B*) are the entropy of *A* and *B*, respectively, and *H* (*A*, *B*) is their joint entropy. In image registration, the entropies are calculated by the probability distributions shown as Eq (3), (4), and (5).

$$H(A)=-\sum_{a\in A}p_{A}(a)\text{log }p_{A}\left(a\right)$$ (3)

$$H(B)=-\sum_{a\in B}p_{B}(b)\text{log }p_{B}(b)$$ (4)

$$H(A,B)=-\sum_{a\in A}\sum_{b\in B}p_{AB}(a,b)\text{log }p_{AB}(a,b)$$ (5)

Here, *a* and *b* are the intensity values in images *A* and *B*, respectively; *p*_*A*_ *(a)* and *p*_*B*_ *(b)* are the marginal probability distribution of *A* and *B*, respectively; and *P*_*AB*_ *(a*,*b)* is the joint probability of *A* and *B*. Corresponding intensities *a* in image *A* and *b* in *B* are related though geometric transformation.

Entropy is a measure of the amount of uncertainty that exists regarding the variable. If the value of a measurement (pixel or voxel in the image) about which the viewer is very uncertain is determined, then a large amount of information is gained. However, if a value has a high probability, then it is easy to guess and only a small amount of information is gained. These properties can be used to show that an image with similar numbers of each intensity value contains more information or has a higher entropy value than an image for which a majority of intensity values have the same value. The joint entropy *H*(*A*, *B*) measures the dispersion of the joint probability distribution. The joint probability distribution should have fewer and sharper peaks when the images are registered than in any case of misalignment. Non-corresponding combinations of *a* and *b* in the overlapping parts will be aligned at misregistration, causing dispersion in the probability distribution, and leading to greater joint entropy value. The mutual information criterion states that the images are geometrically aligned when *MI* is maximal [22].

As the similarity measurement of two images, the principles of the mutual information can be as: the correlation-ship degree of the corresponding feature points of the two images is largest when the two images reach consensus in spatial position after registration.

$$T'={\text{arg}}_{T}\text{max }MI\left(A,T\left(B\right)\right)$$ (6)

Where, *A* is the reference image, *B* is floating image, and *T* is the transformation function.

Base on the Eq (6), it is obvious that medical image registration based on mutual information procedure is essentially a multi-parameter optimization. According to the registration principles of the maximum nonlinear correlation coefficient, we need to find an optimal transformation function T, under which the nonlinear correlation coefficient between the reference image and the transformed floating image is the largest. Apparently, this is the problem of finding the maximum value of the multi-dimensional variables. In this paper, we employed the Powell algorithm [23], which is a searching algorithm divides the searching process into several phases. Each phase consists of *n*+1 one-dimensional search process, and each search process will search the N dimension we have set to get the optimal point. The searching process should along the initial point to the optimal point, and replace the point with the optimal point at one of the dimensions, and then enter the next iteration.

In the Powell algorithm, the *N* orthogonal searching directions ($S_{0}^{1},S_{0}^{2},\cdots ,S_{0}^{n}$) are preliminary given, and the first round of optimization cycle should along the given directions, that is, to get the optimal approximation direction factor of each search direction makes $X_{k}^{\left(i\right)}$ approach the optimal value of the direction.

$$X_{k}^{\left(I\right)}=X_{k-1}^{\left(I\right)}+t\cdot S_{k}$$ (7)

After finishing the first round cycle, we replace the original searching direction *S*_1_ with the difference of the $X_{k}^{\left(i\right)}$ between the beginning and the end of the searching. The following searching process is the same as above until we reach the stop criterion.

#### 2.2.3. Segmentation of SPNs based on an improved watershed algorithm

After the image registration depicted above, the spatial position has reached consensus of the CT and PET images. Thus, we only focused on the segmentation of the suspicious areas on CT images which corresponding with that in PET images.

The watershed algorithm in image segmentation is a method of mathematical morphology segmentation based on topological theory. The basic principle of the algorithm is to model the image as topology landforms in geodesy [24]. The grayscale values of the pixels in the image can be quantified in a manner similar to altitude pixels, and local values in affected areas can be considered collection basins, with the connected boundaries forming a watershed. Suppose the existence of a catchment basin (regional minimum) in a natural area. In this basin, the water rises at a uniform rate. The water gathers in different water basins, blocked by dams, producing a dam watershed as a dividing line. This watershed algorithm is easy to use, and it allows easy parallel processing, but it can easily cause over-segmentation, dividing the image into too many small areas, flooding the target of interest.

With the traditional watershed method, which involves flooding along a direction perpendicular to the plane, the dam is the local maximum of the curve. The resulting boundary of each dam is the set of the local maximum values along their directions. In this paper, a new watershed method was derived based on token-based water basin flooding according to the original watershed method.

The new method was used to identify pulmonary nodules in a CT image; and its results laid the foundation for the extraction of the features of SPN.

Let *M*_1_,*M*_2_,*M*_3_,…,*M*_*g*_ represent the minimum local values of the image *G(x)*. Let *C(M*_*i*_ *)* represent the water basin associated with the minimum area *M*_*i*_. Let min and max represent the minimum value and maximum value of the gradient. Suppose the overflow process to be equivalent to the addition of a gray scale value, with *n* as the additive value of the overflowing process and *T(n)* representing the set of *x* that *f(x)<n*, where *f(x)* is the gradient image signal. When overflow takes place, let *C*_*n*_ *(M*_*i*_ *)* be considered an associate part with the minimum area *M*_*i*_. That is, let *C*_*n*_ *(M*_*i*_ *)* be the binary image where *n* is the overflowing depth and *C(M*_*i*_ *)* is the water basin that forms.

$$C_{n}\left(M_{i}\right)=C(M_{i})\cap T[n]$$ (8)

If the gray value of the minimum area *M*_*i*_ is *n*, then the overflowing part is the same as the minimum area in the (*n+1)th* step, namely *C*_*n*+1_ *(M*_*i*_ *)* = *M*_*i*_. Let *C[n]* represent all the water basins in the *nth* step and let *C [max+1]* represent all the water basins in the overflow process. The details of the steps of the method are as follows:

Step 1: The edge feature points of the image based on the texture are marked.

Step 2: The image is penetrated the image at the marked points in all directions until the local maximum point is found;

Step 3: A dam is constructed at the point of stoppage and the segmentation boundary derived by combining the dams of all directions.

**Fig 2:**
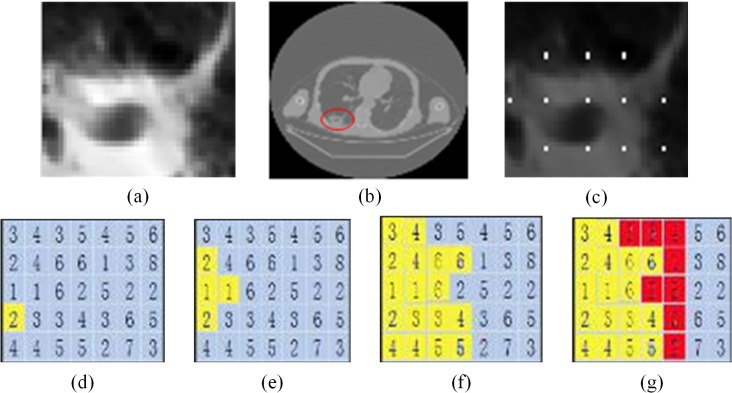
Segmentation of improved watershed algorithm. (a) original SPN image; (b) segmented SPN in the lung; (c) marked points on the SPN; (d), (e), (f), and (g) penetrated areas.

To split the full ROI, this method requires a plurality of seed points in the target area. Eventually, the dam floods the internal dams, leaving only the outermost dams. The final boundaries of the ROI are then determined. Fig 2 shows the segmentation process of the improved watershed algorithm. If the heights of the penetrated areas are less than those of the adjacent water holes, then the water holes begin to leak. To prevent leakage from the water hole, dams are built on the boundaries of the areas. Finally, all the dams connect together and form the boundary of the segmentation.

#### 2.2.4. Feature extraction of solitary pulmonary nodules on PET/CT

Feature extraction transforms an image from a high-dimensional space to a low-dimensional space. The extracted features can be used to describe each image uniquely. The feature extraction method can compress the intrinsic dimensions but it leaves the image symbol attributes unchanged. The CT image displays the tissue density structure of the thorax, in which the differences in density between an SPN and the surrounding parenchyma can be found. The most common method of computing the textural features of an image involves gray-level co-occurrence matrix. If the gray level co-occurrence matrix of the suspicious regions of CT images were calculated, and then the contrast, correlation, energy, homogeneity, and entropy features were calculated based on it [25].

$$CON=\sum_{n=0}^{k-1}n^{2}\left\{\sum_{\left|i-j\right|=n}G\left(i,j\right)\right\}$$ (9)

Eq (7) reflects the clarity of the images; greater values indicate the deeper texture grooves and therefore a clear image.

$$COR=\sum_{i=1}^{k}\sum_{j=1}^{k}\frac{\left(ij\right)G\left(i,j\right)-u_{i}u_{j}}{s_{i}s_{j}}$$ (10)

Eq (8) is the correlation matrix that reflects the consistency of the images. If the horizontal value of the image is larger than that of the vertical then, the image has a horizontal texture.

$$ASM={\sum_{\text{i=}1}^{k}\sum_{j=1}^{k}\left(G\left(ij\right)\right)}^{2}$$ (11)

Eq (9) is the energy that reflects the homogeneity and the thickness of the images. The more uniform is the distribution of the gray areas, the less energy there is.

$$ENT=-\sum_{i=1}^{k}\sum_{j=1}^{k}G\left(i,j\right)\text{log }G\left(i,j\right)$$ (12)

Eq (10) is the entropy that reflects the amount of information in the image. More information indicates more entropy.

Because the gray level co-occurrence matrix can be calculated based on the rectangular area, the ROIs of the suspicious nodules were usually irregular. For this reason, correlation processing is necessary. There are two kinds of common disposal methods: The first involves ROI of the outer rectangle blank area, and it uses a range of mean and median values. The second involves filling the pixels in the original image. Because there are always errors in the segmentation process of registration of suspicious nodules, the second method was selected to reduce error. PET images reflect the level of radiopharmaceutical uptake by different tissues of each living subject. The SUV—usually SUV_max_—is widely used in the detection of lung cancer. In this study, the maximum standardized uptake value (SUV_max_) of the pulmonary tumor was calculated in all cases using a 3-dimensional acquisition and the following formula:

$$SUV_{\text{max}}=\frac{\text{max }imum\;tissue\;concentration(MBq/g)}{injected\;dose(MBq)/body\;weight(g)}$$ (13)

Here, the maximum tissue concentration was represented by the counts per second of the voxel showing the maximum radioactivity in the volume of interest encompassing the tumor divided by the volume of the voxel (mL).

#### 2.2.5. Classification based on SVM

Support vector machine (SVM) is a machine learning method based on the statistical learning theory of Vapnik-Chervonenkis dimension theory [26], and mainly solves the small sample problem. SVM maximizes the margin by determining a separating hyper-plane to identify different classes of data [27, 28]. The radial based function (RBF) is the most widely used kernel function because of its lower computing complexity, which is suitable for the classification of small samples.

As given above, to validate our proposed method of detection, SPNs were classified as malignant or benign based on textural features of CT images and metabolic features (SUV_mean_) of PET images of segmented images using SVM based on RBF. For this work, the textural features of CT images including contrast, correlation, energy, homogeneity, and entropy were evaluated. In the process of classification based on SVM theory, the 10-fold cross validation was used to facilitate good performance. Cross validation is a statistical method used to validate the performance of classifiers. The basic theory of cross-validation is to divide the data into training sets, which are used to train the classifier, and validation sets, which are used to test the classification model. The 10-fold cross validation was used to divide the total data into 10 parts, 9 of which were taken as the training set (197) and 1 of which (22) served as the testing set. It was also used to train and test the classifier 10 times. In order to reduce the complexity of the calculation the classification of the SPNs was implemented based on the 10-fold cross validation.

Here, 219 patients, of whom 120 (54.7%) had possible SPN and 99 (45.3%) had possible inflammation were selected. Two criteria (sensitivity, false-positive rate) were used to evaluate detection performance. SPNs detected by our proposed method and by traditional methods based on SVM are depicted in Table 1. The entire proposed process was used on the same database. A three-phase experiment was used to confirm our proposed method’s performance. First, the segmentation method used was based on features of CT and PET images. Next, we extracted textural features from CT and metabolic features (SUV_max_) from PET. This permitted differentiation of malignant and benign SPNs based on SVM. Finally, we compared traditional methods to our proposed technique.

**Table 1:** Sensitivity and false-positives for traditional and proposed methods.

Criteria	Dehmeshki	Suarez Cuenca	Opfer	Messay	Choi	Proposed Method
Sensitivity (%)	90	80	75	85	93	95.6
False Positive (%)	16	8	5	2.8	3.5	2.9

## Results

**Fig 3:**
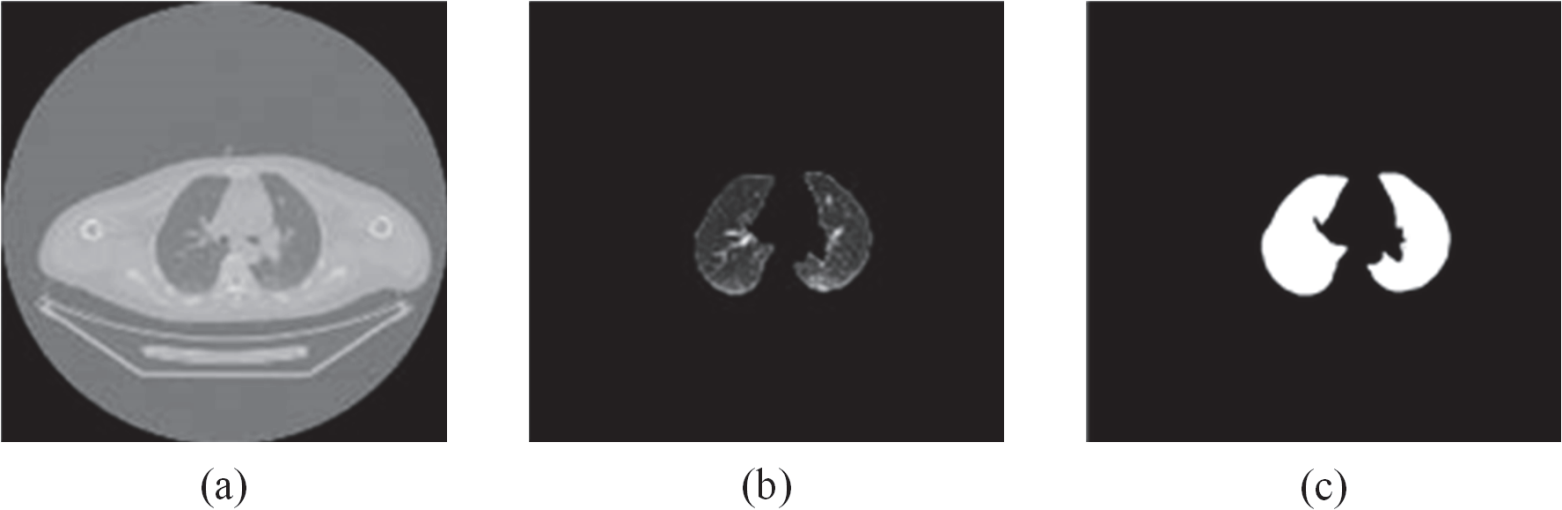
Segmentation as produced by the proposed method. (a) original image; (b) results of segmentation; (c) binarized lung parenchyma.

**Fig 4:**
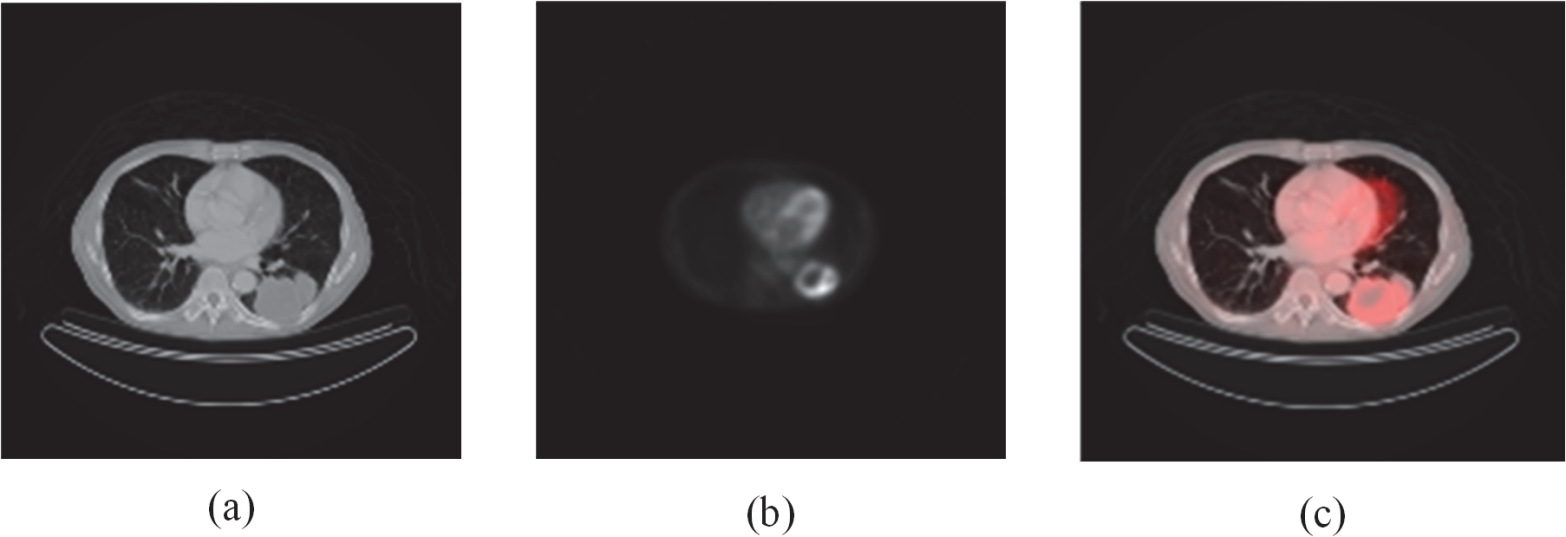
Results of PET/CT image registration. (a) CT image; (b) PET image at the same position; (c) results of image registration.

**Fig 5:**
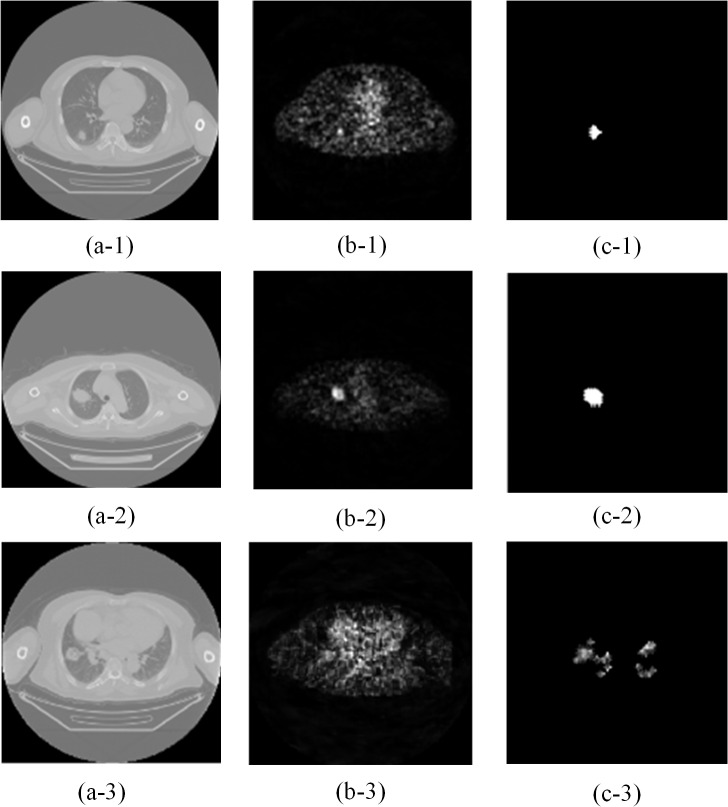
Results of nodule segmentation in different PET/CT images. (a-1, -2, and 3) CT images; (b-1, -2, and 3) corresponding PET images; (c-1, -2, and 3) results of segmentation.

Fig 3 shows lung segmentation in a CT image and Fig 4 depicts results of PET/CT image registration. Fig 5 displays data from nodule segmentation in PET images and data show that after the segmentation of the suspicious region in the PET image, there was only one focus in (c-1) and (c-2), and there were four suspicious foci in (c-3) according to the c series images.

**Fig 6:**
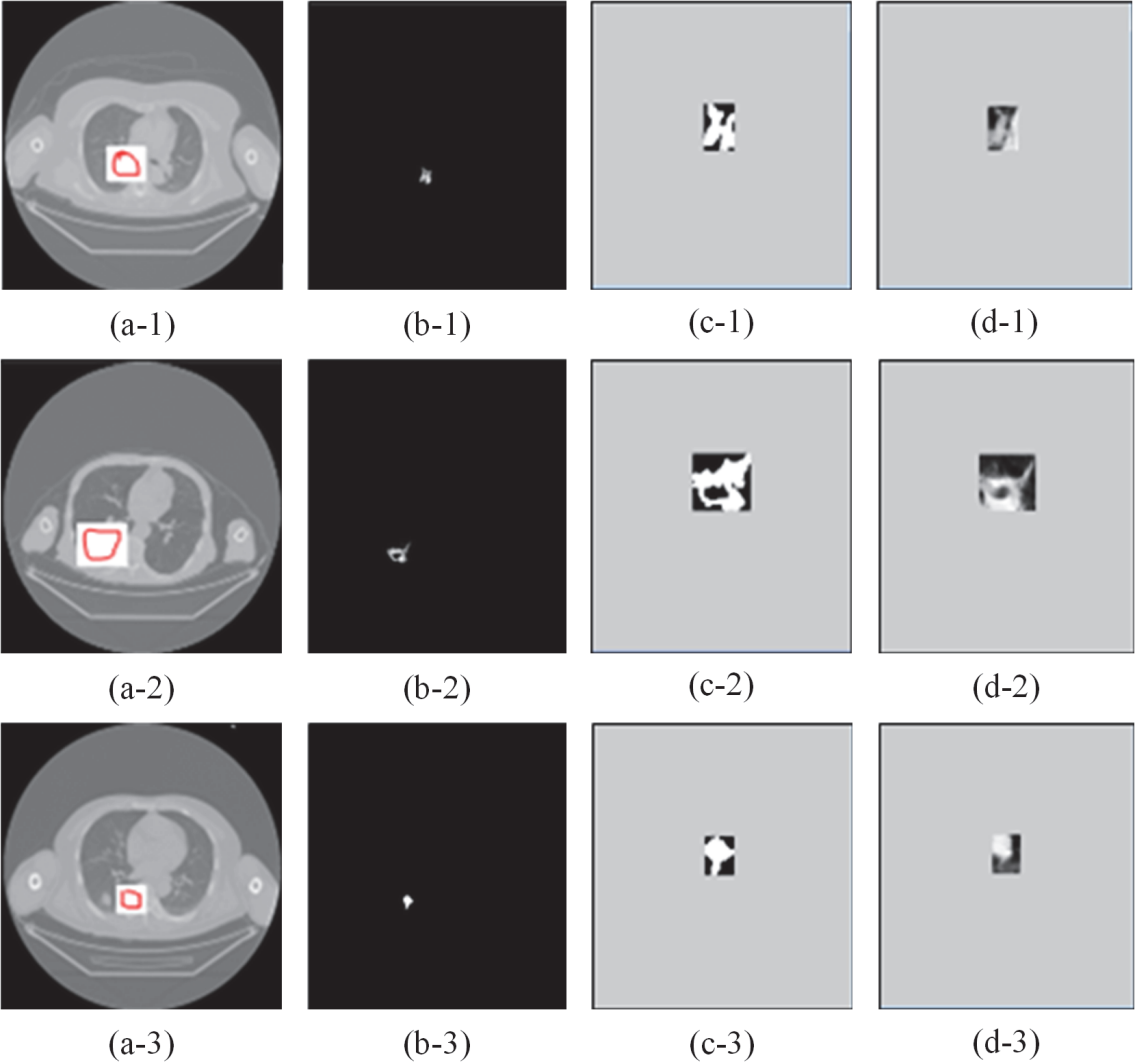
Segmentation results of suspicious nodules in CT images. (a-1, -2, and 3) suspicious nodule areas; (b-1, -2, and 3) suspicious nodules evaluated by a dynamic threshold segmentation method; (c-1, -2, and 3) show the resulting outer rectangle templates; (d-1, -2, and 3) show the suspicious nodules with the outer rectangles.

Fig 6 shows segmentation results of suspicious nodules in CT images. To evaluate the performance of our proposed SPN segmentation, current traditional methods were compared to segmentation results of our proposed method with manual segmentation methods. Table 2 depicts these data and indicates that our proposed method offers good segmentation performance. The last column of Table 2 depicts segmentation results that confirm greater accuracy for segmentation than a method based on a growing region and dynamic threshold.

**Table 2:** Accuracy of pulmonary nodules segmentation.

Methods	APNSL	PNSLM	ONPMS	SAR
Proposed method	944	984	927	94.2%
Region growing	1087	984	903	91.8%
dynamic Threshold method	891	984	876	89.1%

APNSL: average pixels of segmented lesions; PNSLM: pixels in segmented lesions based on the manual; ONPMS: number of overlapping pixels with manual segmentation; SAR: accuracy of segmentation.

To distinguish nodules more clearly, textural features such as contrast, correlation, energy, homogeneity, and entropy of the nodule were selected. Table 3 presents nodules and non-nodule traits, including contrast, correlation, energy, homogeneity, entropy, and SUV_max_. In Table 3, *contrast* refers to the differentiation of the adjacent pixels and the clarity and the depth of the groove of image texture. A larger value indicates a greater groove depth. *Correlation* refers to the consistency of the image texture; if the image has a texture running horizontally, the value of the horizontal direction will be larger than that of the vertical direction. *Energy* refers to the uniformity of the gray distribution and the coarseness of the texture. *Homogeneity* refers to the degree of grayscale value distribution in the image. *Entropy* refers to the degree of the complexity and the non-uniformity of the texture in image. *SUV*_*max*_ refers to the maximum uptake by the nodule related to background, correcting for body weight. A higher value indicates a higher probability of malignancy or inflammation.

**Table 3:** Characteristics of nodules and non-nodules.

Area Feature	Nodule 1	Nodule 2	Nodule 3	Non-nodule 1	Non-nodule 2	Non-nodule 3
Contrast	1.0957	2.9471	0.9457	1.1111	0.9776	1.3558
Correlation	0.8844	0.6846	0.9296	0.6635	0.4264	0.6598
Energy	0.0767	0.0634	0.0929	0.3795	0.1982	0.1980
Homogeneity	0.7375	0.6304	0.7572	0.8058	0.7595	0.7383
Entropy	3.1273	3.3008	2.8753	1.9750	2.1980	2.4573
SUVmax	7.1563	3.2326	2.7012	2.4304	2.5316	2.3694

**Fig 7:**
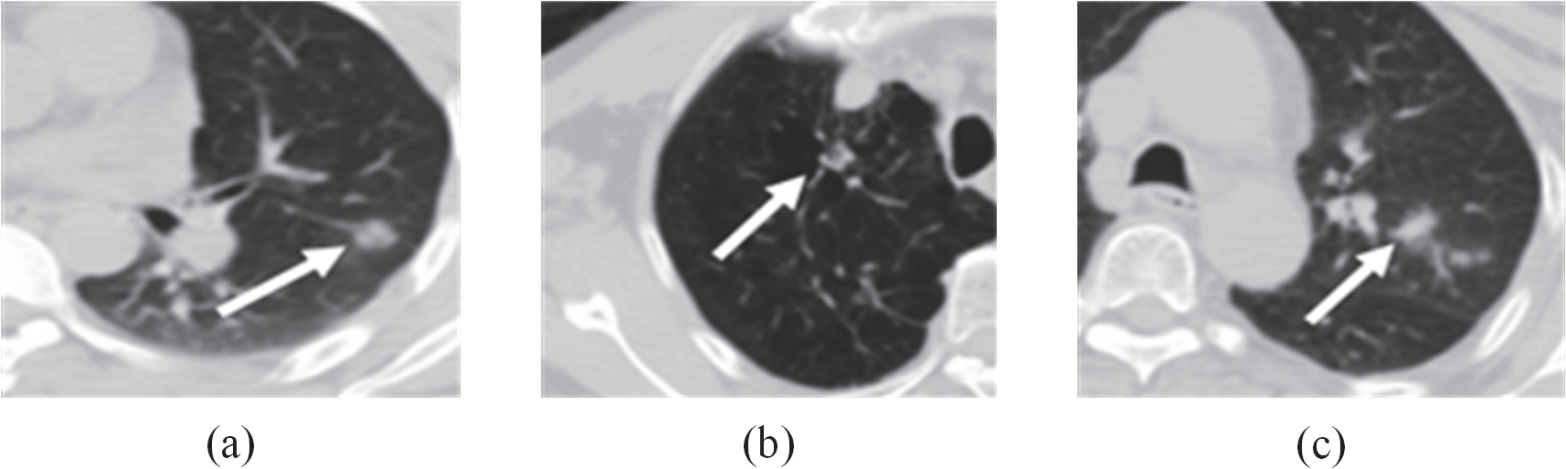
Low-contrast nodules detected by the proposed method.

**Fig 8:**
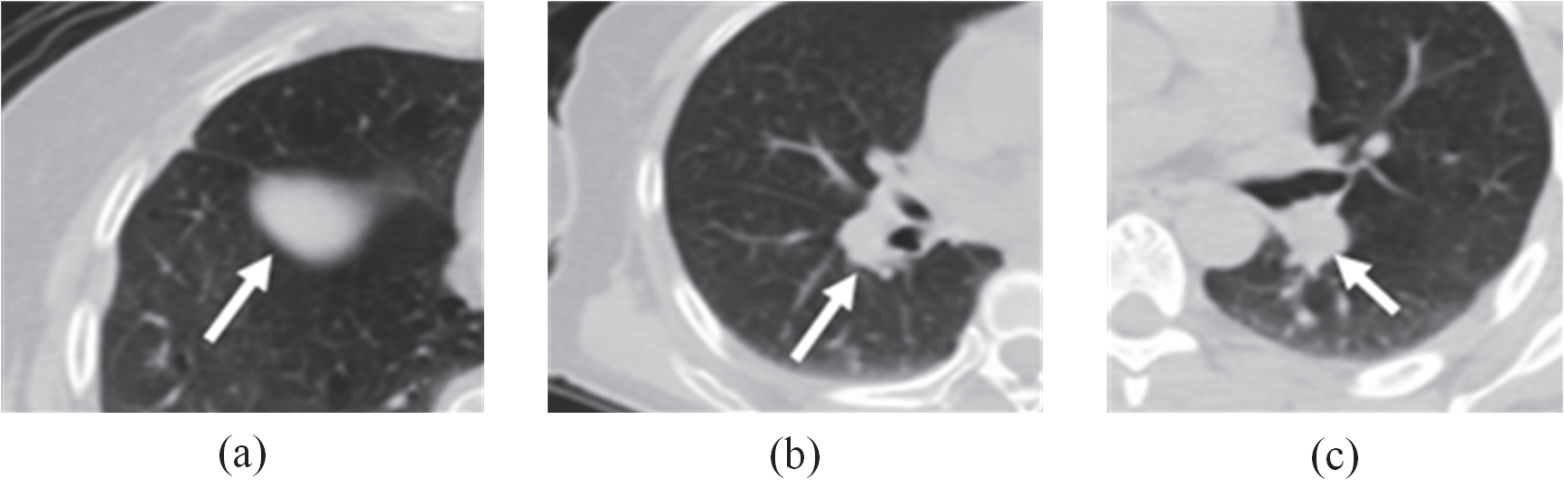
Non-nodules correctly detected based on the proposed method.

Table 1 depicts traditional and proposed method sensitivity and false positives. Figs 7 and 8 show examples of the nodules and non-nodules detected based on the proposed methods and these were better than nodule detection depicted in Table 1. Sensitivity can be improved by 2.6% (from 93% based on the Choi method), and the rate of false positives was relatively low. In this paper, traditional methods were based on CT image features images, which affected low contrast nodule detection accuracy. Low contrast nodules are shown in Fig 7. Methods based solely on CT features always failed to detect them. Because CT features of those nodules were ambiguous, gray level-based traditional methods could hardly detect them. PET images depict tumor metabolic features, so our proposed methods can account for nodular metabolic features (SUV_max_) that reflect tumor traits.

**Fig 9:**
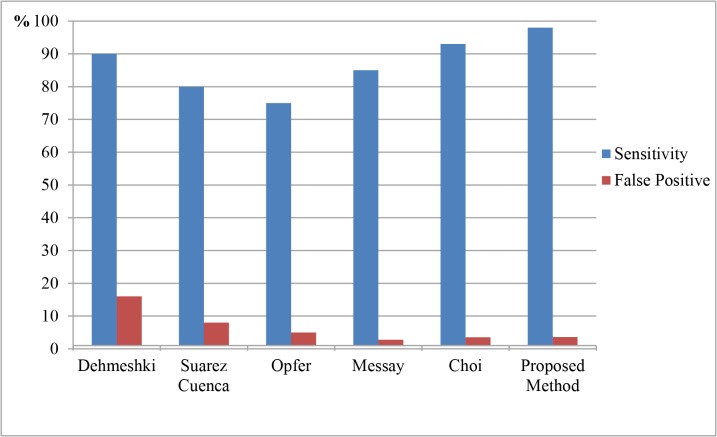
Comparison of the traditional methods based on SVM.

Blood vessels and non-nodules (nodules with inflammation) always had similar CT features compared to nodules. Table 1 shows that traditional method false-positives were relatively high and most were comprised of vessels and inflammation. Traditional methods are mainly based on nodular CT features and thus may misdiagnose vessels or non-nodule areas as SPNs. Moreover, these regions in PET images do not always have pronounced metabolic features. In contrast, our proposed method can differentiate SPNs from vessels and non-nodule areas more effectively. Fig 8 shows that traditional methods reviewed here misidentified these areas as SPNs, but our proposed method identified them as vessels or nodules with inflammation. Table 1 and Fig 9 confirm that our proposed methods are more sensitive than traditional methods, and that they offer better detection. We had the fewest false-positives (2.9/scan) than other methods and our sensitivity was not approached by the traditional methods reviewed here.

## Conclusion

Our work indicates that our proposed method can distinguish malignant from benign SPN to assist with differential diagnosis using both SPN traits as detected by CT and PET. This method is more sensitive and accurate than CT or PET alone or any other traditional methods. To our knowledge, this is the first report of its kind. Our method is also less time consuming, compared to manual segmentation by two observers. Even the recently available semi-automated software can segment SPN-based CT images in few seconds, but these CT images cannot account for metabolic traits detected in PET images which offer better diagnostic accuracy. Our proposed method accounts for the textural traits of CT images as well as metabolic characteristics of PET which increases its sensitivity and decreases the number of false-positives compared with CT or PET alone or traditional methods. Our method has been combined with semi-automated software, and these tests are currently underway. Present data suggest that semi-automated software and our method offers good results without systematic errors. Our current study was limited by the lack of a general evaluation standard as well as a small SNP sample size. This prevented analysis of smaller subdivisions, which affects the generalization of our proposed method. Identifying more SPNs characteristics with larger experiments is needed to reduce false-positives for vessels and non-solid nodules and these efforts will be undertaken in future studies.
